# Examining the Prevalence, Characteristics, and Potential Links Between Skin Disorders and Autism Spectrum Disorder (ASD)

**DOI:** 10.3390/jcm14020469

**Published:** 2025-01-13

**Authors:** Laila Yousif Al-Ayadhi, Nadra Elyass Elamin, Abdulaziz Madani, Farah Al-Ghamdi, Hend Ali Al-Ghamdi, Dost Muhammad Halepoto

**Affiliations:** 1 Department of Physiology, College of Medicine, King Saud University, Riyadh 11451, Saudi Arabia; lyayadhi@ksu.edu.sa; 2 Autism Research and Treatment Center, Department of Physiology, College of Medicine, King Saud University, Riyadh 11461, Saudi Arabia; hendalghamdi112@gmail.com (H.A.A.-G.); dr_m_halepota@yahoo.com (D.M.H.); 3 Department of Dermatology, College of Medicine, King Saud University, Riyadh 11451, Saudi Arabia; amadani1@ksu.edu.sa; 4 College of Medicine, Dar Al Uloom University, Riyadh 13314, Saudi Arabia; farahalghamdi1999@gmail.com; 5 Prince Sultan Medical Military City, Riyadh 12233, Saudi Arabia

**Keywords:** autism spectrum disorder, skin disorder, atopic dermatitis, eczema, dry skin, allergies

## Abstract

**Background:** Increasing evidence indicates that skin disorders may contribute to an increased risk of autism spectrum disorder (ASD). They can affect the quality of life, and they have an impact on social isolation, behavioral problems, cognitive scores, and some subscales of ASD. **Methods:** This study was an online questionnaire-based, observational, and cross-sectional study conducted during the period from August 2022 through January 2023 to examine dermatological manifestations among ASD individuals compared to controls. Descriptive and non-parametric tests were used for data analysis. **Results:** A total of 363 individuals with skin diseases were interviewed during the study period. In total, 189 (52.1%) of participants were autistic and 174 (47.9%) were controls. Asthma, anxiety, depression, and autoimmune disease were persistent in the ASD group compared to the controls (*p* < 0.001). The results also show that skin, food, and respiratory allergies were statistically significantly associated with ASD (50%, 22.2%, 14.8%, respectively) compared to the controls (26.4%, 10.3%, 7.5%, respectively) (*p* < 0.05). The most prevalent disease in the controls was eczema (15.5%), followed by dry skin (14.9%) and acne (10.3%). These diseases showed a statistically significant association with ASD compared to the controls (*p* < 0.0001). **Conclusions:** Our findings indicate that atopic disorders and comorbidities, including eczema, asthma, and allergies, are significantly associated with ASD. A large population-based study is warranted to clarify the prevalence of skin disorders among individuals with ASD, coupled with the study of the association between skin disorders and comorbidities to determine the relationship precisely.

## 1. Introduction

Autism spectrum disorder (ASD) is a neurodevelopmental disorder characterized by deficits in social interaction, language, and communication, as well as the presence of restricted repetitive behaviors. ASD often co-occurs with various neurodevelopmental disorders, including intellectual disability (ID), attention-deficit hyperactivity disorder (ADHD), epilepsy and seizure disorders, and sensory processing disorders [1]. The etiopathology of ASD includes genetic and environmental risk factors as well as immune system dysfunction. Respiratory, skin, and food allergies are the most common immunological dysfunctions represented in ASD children [2,3,4].

Dermatological disorders are mostly comorbid with ASD. Several studies have reported a significant increase in the global prevalence of dermatological disorders among ASD children. They found a significant correlation between ASD and skin disorders, which is attributed to the shared genetic and environmental pathways [4,5,6]. They can affect the quality of life; they impact social isolation, behavioral problems, cognitive scores, and specific subscales of ASD [7,8,9]. Atopic dermatitis (AD) and eczema are the most common skin disorders in ASD subjects [5,10]. Increasing evidence indicates that skin abnormalities may contribute to an increased risk of neurodevelopmental disorders, starting early during childhood [5,11]. The intensity of behavioral problems is significantly associated with disease severity [12,13,14,15]. This is also correlated with an increased level of irritability, social withdrawal, hyperactivity, stereotypy, altered sensory sensitivity, and reduced functional outcomes in ASD subjects [4,12,16]. Increasing evidence indicates that atopic diseases have been considered as potential modulators of neurodevelopment [17]. Allergic conditions such as allergic rhinitis, food allergy, skin allergy, and asthma are highly prevalent among ASD subjects compared to controls [7,10,15]. However, skin disorders underlie the rise in allergic and autoimmune chronic conditions [18]. Furthermore, some dermatological conditions may develop as a consequence of repetitive or stereotyped behaviors and self-injurious behaviors in ASD subjects, such as hand flapping, hand squeezing, hair pulling, or skin rubbing [19].

Different studies have reported different outcomes, with minor differences in the prevalence of the reported diseases. Moreover, subgroup analyses have shown significant differences between age groups and gender in the prevalence of skin diseases. Several explanations for the incidence and prevalence variation have been proposed, including geographical, genetic, climatic conditions, hygienic standards, and socio-economic status [20,21,22,23,24].

The prevalence of skin disorders in the Saudi population was approximately 23.6%. Atopic dermatitis was the most common form of eczema and the most common skin disease [20,22,25]. A wide range of skin disorders was observed among children in different regions in Saudi Arabia. The most common diseases are eczema, acne, allergy, skin irritation, and lichen planus [21,26]. However, different studies have reported different findings. This variation is likely due to the variation in the geographical regions, study settings, study design, or sample size [20,25,26]. The incidence and the prevalence of skin disorders mainly depend on geographic, ethnic, genetic, dietary, social background, hygiene, and weather conditions [20,27]. A recent genetic study on Saudi children with eczema revealed four new genetic mutations that had not been previously reported in the database [28,29].

To the best of our knowledge, the pattern and distribution of skin disorders in ASD subjects have never been studied in Saudi Arabia. Therefore, this study represents the first attempt to determine the pattern of skin disorders among ASD individuals and compare it with a control group. The management of skin disorders in ASD is challenging, as it may be useful in reducing autistic behaviors and improving functional outcomes in ASD subjects.

## 2. Materials and Methods

### 2.1. Setting

This study was an online questionnaire-based, observational, and cross-sectional study conducted at the Autism Research and Treatment Center, College of Medicine, King Saud University, Riyadh, Saudi Arabia, from August 2022 through January 2023 to examine the dermatological manifestations among ASD individuals compared to the controls.

### 2.2. Study Design

A self-administered online questionnaire consisted of 26 items divided into four sections: the first section covered the sociodemographic data, the second section covered the participants’ medical history and any medications used, the third section reviewed types of diet and allergies, and the fourth section focused on skin diseases and other skin-related issues.

The investigators developed the questionnaire after extensive review of the current literature, which was then reviewed and validated by a consultant dermatologist. A pilot study was carried out on a small group to ensure the reliability of the questionnaire’s content. The questionnaire was disseminated via web link through various social media platforms, such as WhatsApp and Twitter. All participants or their caregivers completed the questionnaire to provide a comprehensive medical background of the participants to identify pre-existing conditions and comorbidities, including sociodemographic data, diagnosis of ASD or any skin disorders, treatments, and psychological aspects, such as anxiety and depression.

Before enrollment in the study, informed consent was obtained from each participant. The questionnaire was filled by the participant, their parent/guardian, or their caregiver.

### 2.3. Ethical Approval

Ethical Approval was obtained from the Institutional Review Board, College of Medicine Research Centre, King Saud University, Riyadh, Saudi Arabia, according to the most recent Helsinki declaration (Approval number: 22/0469/IRB on 20 January 2022).

### 2.4. Data Analysis

The data were analyzed using the Statistical Package for the Social Sciences (SPSS) version 26 (IBM Corp., Chicago, IL, USA). Normal distribution of the variables was tested using the Shapiro–Wilk test. The results were summarized and reported as frequency (n) and percentage (%) for all categorical variables. A chi-square test was performed to measure the association between skin disorders and ASD and compare this with the control group. *p* ≤ 0.05 was considered to be significant.

## 3. Results

### 3.1. Sociodemographic Characteristics of Study Participants

A total of 363 individuals with skin diseases were interviewed during the study period, representing a 100% response rate.

Table 1 presents the summary of the sociodemographic data of the participants. Among the 363 respondents, 189 (52.1%) were autistic and 174 (47.9%) were controls. More than 80% of the respondents in the two groups were from Riyadh. Males comprised 87.3% of the ASD group, while females were most prevalent in the control group (58%). Further, 24.9% of respondents in the ASD group were aged between 6 and 10, whereas 27% of respondents in the control group were aged between 11 and 15. Most respondents were Saudi: 168 (88.9%) were in the ASD group, and 137 (78.7%) were in the control group.

There was a remarkable variation in the educational level among the respondents. Training centers were reported as the most frequent education level among ASD (45%), followed by no school (20.6%) and special schools (19%). In contrast, the most frequent education level in the control group was primary school (35.1%), followed by university (21.8%) and secondary school (17.8%). Most of the respondent’s parents were employed (>70%). The reported average monthly income of the families was between SAR 20,001 and 40,000 in the ASD group and between SAR 10,001 and 20,000 in the control group.

### 3.2. Medical History of the Study Participants

Table 2 presents a detailed comparison of the medical history and medication usage of the participants in both groups. Comorbidities were observed in approximately two-thirds of the ASD group and less than 30% in the controls. The most common diseases among the ASD group were asthma (33.3%), anxiety (32.3%), depression (22.8%), and autoimmune disease (21.7%). They were significantly more persistent in the ASD group compared to the controls. The chi-square test result revealed a significant association between these diseases and ASD, compared to the controls (*p* < 0.001), indicating a strong statistical relationship. We observed that the majority of the ASD group were taking medications to manage their symptoms and comorbid conditions. For instance, anti-anxiety medications (20.1%), anti-depressants (12.7%), anti-inflammatories (10.1%), and bronchodilators (7.9%) were the most commonly used treatments, which may support the association of ASD with these comorbidities. On the contrary, 73.6% of the control group had no medical history, and only 10% were taking medications.

### 3.3. Diet and Type of Allergies

Table 3 outlines the type of daily diet and allergies. The majority of the respondents were on a normal diet in the two groups, 91% and 93.7% in ASD and control group, respectively. The results show that 50% of ASD subjects had a skin allergy, followed by food allergy (22.2%) and respiratory allergy (14.8%). In contrast, only 26.4% had a skin allergy in the control group, followed by food allergy at 10.3%, and respiratory allergy (7.5%). These allergies were statistically significantly associated with ASD compared to the controls (*p* < 0.05). In the ASD group, the most common triggers for skin allergy were soap/shampoo (34.4%), insect bites (25.9%), pets (25.9%), and pollen grains (20.1%), while in the controls, the most common triggers were soap/shampoo (11.5%), insect bites (11.5%), and food/dairy products (6.3%). Furthermore, fruits and nuts were the most common allergens responsible for food allergies in the study population.

### 3.4. Association of Skin Disorders with ASD Compared to the Control Group

Table 4 demonstrates skin conditions and the distribution of skin disorders among the ASD and control groups. Of the ASD subjects, the most common skin type was sensitive skin (37.6%), followed by normal skin (37.3%) and dry skin (22.2%). More than 50% of the controls had normal skin, 28.7% had dry skin, and only 9.2% had sensitive skin.

With regard to skin diseases, we found that (42.3%) of ASD individuals suffered from dry skin, followed by pruritis (33.9%), eczema (30.7%), and itching (22.2%). Furthermore, the most prevalent disease in the controls was eczema (15.5%), followed by dry skin (14.9%) and acne (10.3%). These diseases showed a statistically significant association with ASD compared to the controls (*p* < 0.0001). Notably, the majority of individuals with eczema also presented dry skin and itching in a subgroup of ASD subjects.

Moreover, as depicted in Table 4, the results also revealed additional information about the treatment of skin diseases, the use of skin care products, and the effect of exposure to the sun without protection. We observed that the most commonly used treatments among the participants were topical treatments. Corticosteroids were used in 28.5% of the ASD group compared to 9.2% of the controls, whereas 12.2% of the ASD group used isotretinoin compared to 2.3% of the controls. More than two-thirds of the ASD group were affected by exposure to the sun, compared to less than 50% of the controls.

**Figure 1:**
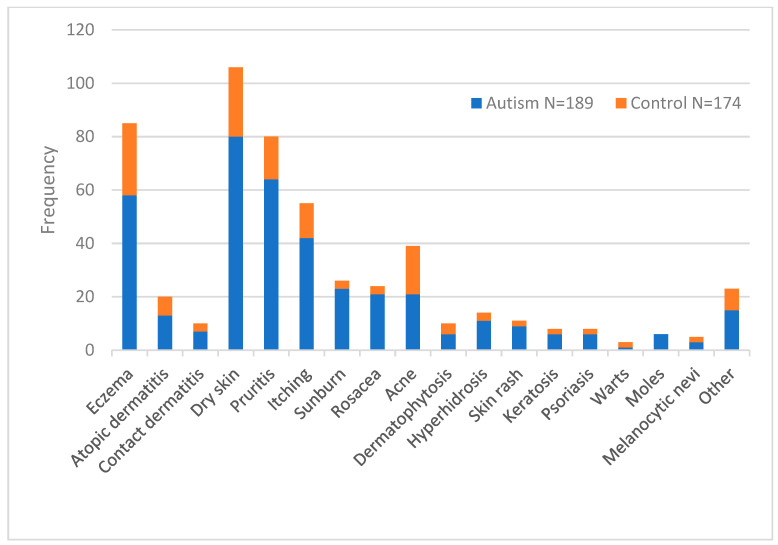
Distribution of skin disorders in ASD and control groups.

Table 5 and Figure 1 show the association of skin disorders with ASD in the study groups. The prevalence of eczema, dry skin, pruritis, itching, sunburn, rosacea, hyperhidrosis, and moles was statistically significantly higher in the ASD group compared to the controls (*p* < 0.001, 0.05, respectively). Moreover, no significant difference between skin diseases and age was observed in the study groups. When we pooled our samples (n = 363), we observed a significant difference in the prevalence of eczema (*p* = 0.18), dry skin (*p* = 0.03), and pruritis (*p* = 0.02) across the five age groups (*p* < 0.05), as represented in Table 6.

## 4. Discussion

ASD is commonly associated with skin disorders and other comorbidities. ASD often co-occurs with various neurodevelopmental disorders, including intellectual disability (ID), attention-deficit hyperactivity disorder (ADHD), epilepsy and seizure disorders, and sensory processing disorders [1]. These comorbidities complicate the diagnosis and treatment of ASD and are associated with the severity of the disease. Therefore, it is crucial to consider them in the assessment and treatment of ASD. In addition, difficulty in communication and sensory processing may play a role in self-stimulating behaviors such as skin picking or scratching. Several studies report that ASD subjects have a high prevalence of skin disorders [4,30]. They shared possible pathophysiological pathways, genetics, environment, alteration in proinflammatory cytokines, microbiota change, and abnormal sensory dysfunction as potential factors [5,6,10].

The frequencies of skin disorders reported in this study varied significantly between ASD and control groups. In the present study, the most prevalent skin disease in the ASD group was dry skin (42%). It was highly statistically significant compared to the controls (7.2%) (*p* < 0.0001). This finding is in line with recent studies that showed children with ASD exhibited an increased transepidermal water loss rate (TEWL) due to dry skin and epidermal dysfunction [31,32]. This finding could be attributed to the effects of multiple factors, including climatic factors, poor skincare routines, and the nature of the skin itself. Notably, our results reveal that more than two-thirds of the participants in the two groups did not use moisturizing creams, which may make the skin more prone to severe skin conditions such as dermatitis, dryness, and itching. In light of this, the greater severity of dry skin in individuals with ASD compared to those without ASD may suggest that an underlying connective tissue abnormality may play a role in the pathophysiology of ASD.

Eczema and atopic dermatitis are complex diseases linked with genetic predispositions and the gut microbiota. Genetic predisposition plays a vital role in immune dysfunction and might be responsible for some types of atopic dermatitis. Several studies have that eczema is the most common skin disorder in Saudi Arabia. It is widespread among Saudi children [21,22,24].

Strong research evidence has showed a potential link between eczema and ASD. Children with ASD are more likely to have psoriasis, eczema, and allergies than neurotypical children. The prevalence of eczema among children with ASD ranges from 7 to 64.2% [4,7]. Recent studies reported a significant correlation between eczema and an increased severity of autism symptoms in children with ASD; they attributed this link to the shared embryonic origin of skin and brain tissue. They also suggest that extreme sensory sensitivity is a mediator of worsening behavioral outcomes and may be linked to increased cutaneous discomfort [5,15,33].

In our study, eczema was significantly higher in the ASD group (33.9%) compared to the controls (15%) (*p* < 0.0001), which is consistent with the results of previous studies in different regions in Saudi Arabia, which reported a range between 12% and 26% [9,20,22,26,27]. The prevalence of eczema in the control group was consistent with the results of previous studies in Saudi Arabia, but it was higher in the ASD group. This could be due to exposure to allergens in food and environment, genetics, and skin sensitivity. However, the prevalence of eczema ASD in the current study is consistent with previous studies. The severity of ASD symptoms has been implicated in the severity of eczema [4,6,15,33]. However, the severity of the ASD symptoms was unknown among the ASD group, so we were not able to confirm whether this finding was due to ASD symptoms or other factors.

Given that patients with ASD often experience dermatitis/eczema, a significant increase in itching and pruritis in ASD was expected. In the present study, the prevalence of itching and pruritis was significantly higher in the ASD group compared to the controls (*p* < 0.0001). A recent study highlights a possible link between ASD and itching. They reported the contributing role of contactin-associated protein 2 (CNTNAP2) in pain hypersensitivity and itching behaviors in ASD. This may be associated with the severity of ASD symptoms, sensory sensitivity, and higher levels of anxiety, which are hallmarks of ASD [34]. Difficulty in communication and sensory processing may play a role in self-stimulating behaviors such as skin picking or scratching.

Moreover, subgroup analyses showed no significant difference between skin diseases and age groups in the two groups. When we pooled our samples (n = 364), we revealed a significant difference in the prevalence of eczema, dry skin, and pruritis across the five age groups (*p* < 0.001). This finding is consistent with previous studies that reported the association between eczema and younger patients [21]. Another key finding in our study was that eczema was more frequent in individuals with sensitive skin and dry skin and coupled with dry skin and itching in a subgroup of ASD subjects. In light of these findings, we suggest a contributing role of multiple factors in the development of eczema in a subgroup of ASD subjects. A higher prevalence of eczema could also be secondary to the high frequency of peritus, itching, dryness, and skin sensitivity [35].

### 4.1. Association of Atopic Comorbidities and ASD

Accumulating evidence indicates that atopic comorbidities (asthma, rhinitis, food allergy, atopic dermatitis) are strongly correlated with neurodevelopmental disorders [7]. They have been proposed as a potential modulator of neurodevelopment [36]. The prevalence of atopic diseases was significantly increased among ASD subjects [10,15]. They were substantially associated with the severity of ASD outcomes [8,13,15].

Strong research evidence has suggested a significant link between asthma and ASD. It has been demonstrated that children with asthma may have a higher risk of developing ASD, compared to those without asthma. Researchers have demonstrated that children with ASD and other developmental disorders are more than twice as likely to have asthma than the general population [37]. In contrast, Kuo et al. 2020 reported that rhinitis, asthma, and atopic dermatitis were not associated with the cognitive profile of ASD children [36]. This inconsistency may be attributed to different factors, including genetic and environmental factors. The current study showed that asthma was reported in 63 (33.3%) of the ASD group, which is higher than the prevalence of asthma in the general population [38]. Similarly to previous studies, the prevalence of asthma in the ASD group was significantly higher than in controls (9.2%), *p* < 0.0001. This could be attributed to the exposure to allergen substances and climatic weather [37,38,39].

Allergic conditions, including respiratory, skin, and food allergies, were reported as a consequence of immunologic dysfunction in ASD. Our study revealed that skin allergy, food allergy, and respiratory allergy were significantly associated with the ASD group compared to the control group (*p* < 0.0001). These findings are consistent with previous studies, which confirmed a link between those conditions and an increased risk for ASD [4]. In the current study, the prevalence of food allergy was 22.2% in ASD and 10.3% in the controls (*p* = 0.002), compared to 11.25% in ASD and 4.25% in controls in previous studies, where they showed that children with food allergies are more than twice as likely to have autism spectrum disorder than children without allergies [4,7].

Furthermore, we reported that 14.8% of the ASD group had respiratory allergies compared to 7.5% without ASD (*p* = 0.027), and 50.3% of the ASD group had skin allergies, compared to 26.4% without ASD (*p* < 0.0001). These findings are consistent with Xu et al. 2018, who reported similar findings: 18.7% with ASD had respiratory allergies compared to 12% without ASD, and 16.8% with ASD had skin allergies, compared to 9.8% without ASD [4]. Our findings support the notion of the presence of shared mechanisms early in life, which could affect brain development and social functioning, leading to the development of ASD. Inconsistent with previous findings that the association between food allergy and ASD was more robust than respiratory or skin allergy with ASD, we found that the association between skin allergy and ASD was more substantial than that of respiratory or food allergy with ASD. This may be attributed to exposure to allergen substances or climatic weather, or to the population’s demographic characteristics [4].

Taken together, subjects with ASD were more prone to asthma and allergies. These conditions could trigger immune system dysregulation and the release of inflammatory mediators. This may lead to increased irritability and poorer functional outcomes in ASD and may subsequently result in the presentation of some skin diseases in a subgroup of ASD subjects.

The prevalence of depression and anxiety was highly significant in patients with skin disorders [9,40,41]. Several authors have demonstrated that the prevalence of depression among patients with skin disorders was 15.8%, and symptoms associated with skin diseases may increase the risk of depression [24]. These diseases may develop due to chronic itch, sleep disturbance, and neuroinflammation disturbance [23,24,33]. Previous studies have revealed that depression and anxiety disorders are significantly higher among patients with ASD worldwide (30%, 42%, respectively) [34,42]. They are significantly correlated with the core symptoms of ASD, such as repetitive behaviors [7,8]. Children with ASD may exhibit various behaviors such as hand flapping and twisting, hair pulling, and skin scratching that could eventually cause skin conditions [19]. In light of these findings, the significantly higher prevalence of depression and anxiety among the ASD group in this study, compared to the controls, may be related to the core symptoms of ASD, such as stereotyped behaviors. It is also possible that some symptoms associated with skin diseases may cause profound psychological problems and increase the risk of depression and anxiety.

Surprisingly, a low frequency of infectious skin diseases was reported in the current study (<2%), with no significant difference between the study groups, in contrast to previous studies. Those disorders are more likely linked to poor hygiene, low education and socioeconomic status, and environmental conditions [43,44,45]. Therefore, this finding may reflect a good knowledge and awareness about the diseases, and a high socioeconomic and hygienic status standard in the study population.

### 4.2. Limitations of the Study

This study has some limitations. First, the sample size was relatively small, limiting the generalizability of the findings. Second, it was cross-sectional study, which limits the ability to draw causal inferences. Third, data collection was based on self-reported questionnaires, which are subject to biases such as recall bias and inaccurate reporting. Fourth, diagnoses of ASD and skin disorders were not independently verified through clinical assessments or medical records. Therefore, the results reported should be interpreted with caution. Future longitudinal studies with larger samples are warranted to clarify the prevalence of skin disorders among individuals with ASD, coupled with the study of the association between skin disorders and comorbidities to determine the relationship precisely.

## 5. Conclusions

This study, to our knowledge, is the first to examine skin disorders in individuals with ASD in Saudi Arabia. The current study detected significant differences in skin diseases between ASD and the controls, consistent with previous studies. Our findings also indicate that atopic disorders and comorbidities, including eczema, asthma, and allergies, are significantly associated with ASD. Some of them are associated with the pathogenesis of the disease, and others are the consequence of ASD symptoms. Therefore, the identification and treatment of such comorbidities help in the management of children with AD.

Our data suggest that the interaction between ASD, skin diseases, and comorbidities is complex, and there are probably multiple factors and pathways involved. It is unknown whether these conditions co-occur due to shared mechanisms or exposures, or if the development of one of these conditions increases the risk of the other. Therefore, a large population-based study is warranted to clarify the prevalence of skin disorders among individuals with ASD in further detail and to include a wide range of skin disorders, coupled with the study of the association between skin disorders and comorbidities to determine the relationship precisely. This may help in the development of targeted assessment and therapeutic interventions. Moreover, additional investigation designed to examine the association of skin diseases with a particular focus on the severity of ASD is warranted.

## Figures and Tables

**Table 1:** Demographic data of ASD and control participants.

Characteristics	Autism N = 189 (%)	Control N = 174 (%)
**Gender**		
Male	165 (87.3)	73 (42.0)
Female	24 (12.7)	101 (58.0)
**Age group (year)**		
Less than 5	40 (21.2)	24 (13.8)
6–10	47 (24.9)	44 (25.3)
11–15	46 (24.3)	47 (27.0)
16–20	35 (18.5)	21 (12.1)
More than 20	21 (11.1)	38 (21.8)
**Nationality**		
Saudi	168 (88.9)	137 (78.7)
Non-Saudi	21 (11.1)	37 (21.3)
**Participants Education**		
No school	39 (20.6)	22 (12.6)
Primary	17 (9.0)	61 (35.1)
Secondary	8 (4.2)	31 (17.8)
High	4 (2.1)	21 (12.1)
University	0	38 (21.8)
Training centers	85 (45.0)	1 (0.6)
Special schools	36 (19.0)	0
**Parents Occupation**		
Employed	136 (72.0)	135 (77.6)
Unemployed	13 (6.9)	2 (1.1)
Retired	38 (20.1)	27 (15.5)
Other	2 (1.1)	10 (5.7)
**Family Monthly Income (SAR)**		
Below 5000	14 (7.4)	15 (8.6)
5001–10,000	47 (24.9)	38 (21.8)
10,001–20,000	46 (24.3)	51 (29.3)
20,001–40,000	50 (26.5)	37(21.3)
40,001–60,000	23 (12.2)	20 (11.5)
Above 60,000	9 (4.8)	13 (7.5)
**Skin Phenotype**		
White	117 (61.9)	117 (61.9)
Brown	68 (36.0)	68 (36.0)
Dark brown	3 (1.6)	3 (1.6)
Black	1 (0.5)	1 (0.5)

**Table 2:** Comparison of medical history and medication usage between ASD and control participants.

	Autism N = 189 (%)	Control N = 174 (%)	*p*-Value
**Does the participant have any of the following?**			
None	73 (38.6)	128 (73.6)	0.000
Asthma	63 (33.3)	16 (9.2)	0.000
Autoimmune disease	41 (21.7)	7 (3.4)	0.000
Depression	43 (22.8)	6 (3.4)	0.000
Anxiety	61 (32.3)	10 (5.7)	0.000
Thyroiditis	7 (3.7)	1 (0.6)	0.042
Gastrointestinal disease	21 (11.1)	6 (3.4)	0.005
Urinary disease	6 (21.7)	2 (1.1)	0.189
Any active infection	1 (0.5)	1 (0.6)	0.953
Other	13 (6.9)	12 (6.9)	0.995
**Does the participant currently or previously take any medicines?**			
None	100 (52.9)	157 (90.2)	
Anti-depressant	24 (12.7)	0	
Anti-anxiety	38 (20.1)	1 (0.6)	
ADHD	19 (10.1)	2 (1.1)	
Inhaled steroids	9 (4.8)	1 (0.6)	
Bronchodilator	15 (7.9)	5 (2.9)	
Antibiotics	8 (4.2)	0	
Antihistamine	10 (5.3)	1 (0.6)	
Thyroxine	2 (1.1)	1 (0.6)	
Anti-diabetic	0	2 (1.1)	
Anti-hypertensive	1 (0.5)	0	
Immunosuppressant	5 (2.6)	2 (1.1)	
Steroids	6 (3.2)	0	
Anti-inflammatory	19 (10.1)	3 (1.7)	
Other	5 (2.6)	4 (2.3)	

**Table 3:** Types of diet and allergies used by ASD and control participants.

	Autism N = 189 (%)	Control N = 174 (%)	*p*-Value
**Normal daily diet**			
Normal	172 (91.0)	163 (93.7)	
Gluten-free diet	10 (5.3)	2 (1.1)	
Low-calorie diet	4 (2.1)	5 (2.9)	
Other	3 (1.6)	4 (2.3)	
**Type of allergy**			
No known allergy	71 (37.6)	109 (62.6)	0.000
Food allergy	42 (22.2)	18 (10.3)	0.002
Skin allergy	95 (50.3)	46 (26.4)	0.000
Respiratory allergy	28 (14.8)	13 (7.5)	0.027
Medication allergy	6 (3.2)	3 (1.7)	0.375
Other	2 (1.1)	2 (1.1)	0.934
**What are the triggers of skin allergy?**			
Food/dairy products	32 (16.9)	11 (6.3)	
Soap/shampoo	65 (34.4)	20 (11.5)	
Cleansing solution	37 (19.6)	10 (5.7)	
Cosmetics	3 (1.6)	4 (2.3)	
Pollen grains	38 (20.1)	8 (4.6)	
Pets	49 (25.9)	8 (4.6)	
Insect bites	49 (25.9)	20 (11.5)	
Medications	27 (14.3)	4 (2.3)	
Latex	28(14.8)	4 (2.3)	
Other	23 (12.2)	23 (13.2)	

**Table 4:** History of skin disease among ASD and control participants.

	Autism N = 189 (%)	Control N = 174 (%)
**Skin condition**		
Normal	71 (37.6)	90 (51.7)
Dry	42 (22.2)	50 (28.7)
Oily	4 (2.1)	15 (8.6)
Sensitive	71 (3.6)	16 (9.2)
Mixed	1 (0.5)	3 (1.7)
**Which skin disease do you have?**		
None	106 (56.1)	131 (75.3)
Eczema	58 (30.7)	27 (15.5)
Atopic dermatitis	13 (6.8)	7 (4.0)
Contact dermatitis	7 (3.7)	3 (1.7)
Dry skin	80 (42.3)	26 (14.9)
Pruritis	64 (33.9)	16 (9.2)
Itching	42 (22.2)	13 7.5)
Sunburn	23 12.2)	3 (1.7)
Rosacea	21 (11.1)	3 (1.7)
Acne	21 (11.1)	18 (10.3)
Dermatophytosis	6 (3.2)	4 (2.3)
Hyperhidrosis	11 (5.8)	3 (1.7)
Skin rash	9 (4.8)	2 (1.1)
Keratosis	6 (3.2)	2 (1.1)
Psoriasis	6 (3.2	2 ((1.1)
Warts	1 (0.5)	2 (1.1)
Moles	6 (3.2)	0
Melanocytic nevi	3 (1.6)	2 (1.1)
Other	15 (7.4)	8 (4.6)
**Do you ever have any of the following treatments?**		
None	128 (67.7)	149 (85.6)
Accutane	11 (5.8)	6 (3.4)
Cortisone cream	54 (28.5)	16 (9.2)
Isotretinoin	23 (12.2)	4 (2.3)
Oral antibiotics	21 (11.1)	5 (2.9)
Hormone replacement therapy	4 (2.1)	0
**Do you take any medication that makes you sensitive to the sunlight?**		
No	172 (91.0)	171 (98.3)
Yes	17 (9.0)	3 (1.7)
**Which skincare products you are currently using?**		
None	129 (68.3)	115 (66.1)
Moisturizing cream	50 (26.5)	49 (28.2)
Moisturizing cream for eczema	6 (3.2)	4 (2.3)
Sunscreen products	6 (3.2)	4 (2.3)
Cortisone creams	10 (5.3)	4 (2.3)
Isotretinoin/retinoids	3 (1.6)	5 (2.9)
**When you exposed to the sun without protection?**		
No effect	66 (34.9)	97 (55.7)
Always burn	7 (3.7)	4 (2.2)
Always burn, sometimes tan	20 (10.5)	9 (5.1)
Always tan	25 (13.2)	37 (21.3)
Sometimes burn, sometimes tan	71 (37.6)	27 (15.5)

**Table 5:** Association of skin disorders with ASD in study groups.

Disease	Autism N = 189 (%)	Control N = 174 (%)	*p* Value
**Eczema**	58 (16.0)	27 (7.4)	**0.001** *
**Atopic dermatitis**	13 (3.6)	7 (1.9)	0.38
**Contact dermatitis**	7 (1.9)	3 (0.8)	0.25
**Dry skin**	80 (22.0)	26 (7.2)	**0.000** *
**Pruritis**	64 (17.6)	16 (4.4)	**0.000** *
**Itching**	42 (11.6)	13 (3.6)	**0.000** *
**Sunburn**	23 (6.3)	3 (0.8)	**0.000** *
**Rosacea**	21 (5.8)	3 (0.8)	**0.001** *
**Acne**	21 (5.8)	18 (5.0)	0.81
**Dermatophytosis**	6 (1.7)	4 (1.1)	0.61
**Hyperhidrosis**	11 (3.0)	3 (0.8)	**0.04** *
**Skin rash**	9 (2.5)	2 (0.6)	0.12
**Keratosis**	6 (1.7)	2 (0.6)	0.11
**Psoriasis**	6 (1.7)	2 (0.6)	0.18
**Warts**	1 (0.3)	2 (0.6)	0.51
**Moles**	6 (1.7)	0	**0.01** *
**Melanocytic nevi**	3 (0.8)	2 (0.6)	0.7
**Other**	15 (4.1)	8 (2.2)	0.86

Pearson chi-square, * significant *p*-value: *p* < 0.05, *p* < 0.001.

**Table 6:** Association between skin disorders and age groups among the participants (N = 363).

Disease	<5 yrs	6–10	11–15	16–20	>20	*p* Value
**Eczema**	21 (5.8)	18 (5.0)	16 (4.4)	20 (5.5)	10 (2.8)	**0.01**
**Dry skin**	27	19 (5.2)	29 (8.0)	18 (5.0)	13 (3.6)	**0.03**
**Pruritis**	20 (5.5)	17 (4.7)	18 (5.0)	19 (5.2)	6 (1.7)	**0.02**

## Data Availability

All data generated or analyzed during this study are available from the corresponding author upon request.
